# Protection of Coronary Endothelial Function during Cardiac Surgery: Potential of Targeting Endothelial Ion Channels in Cardioprotection

**DOI:** 10.1155/2014/324364

**Published:** 2014-07-13

**Authors:** Qin Yang, Cheuk-Man Yu, Guo-Wei He, Malcolm John Underwood

**Affiliations:** ^1^Division of Cardiology, Department of Medicine and Therapeutics, Institute of Vascular Medicine, Li Ka Shing Institute of Health Sciences, and Institute of Innovative Medicine, Faculty of Medicine, The Chinese University of Hong Kong, Hong Kong; ^2^The Chinese University of Hong Kong Shenzhen Research Institute, Shenzhen 518057, China; ^3^TEDA International Cardiovascular Hospital, Tianjin 300457, China; ^4^The Affiliated Hospital of Hangzhou Normal University and Zhejiang University, Hangzhou, Zhejiang 310015, China; ^5^Division of Cardiothoracic Surgery, Department of Surgery, Prince of Wales Hospital, The Chinese University of Hong Kong, Shatin, New Territories, Hong Kong

## Abstract

Vascular endothelium plays a critical role in the control of blood flow by producing vasoactive factors to regulate vascular tone. Ion channels, in particular, K^+^ channels and Ca^2+^-permeable channels in endothelial cells, are essential to the production and function of endothelium-derived vasoactive factors. Impairment of coronary endothelial function occurs in open heart surgery that may result in reduction of coronary blood flow and thus in an inadequate myocardial perfusion. Hyperkalemic exposure and concurrent ischemia-reperfusion during cardioplegic intervention compromise NO and EDHF-mediated function and the impairment involves alterations of K^+^ channels, that is, K_ATP_ and K_Ca_, and Ca^2+^-permeable TRP channels in endothelial cells. Pharmacological modulation of these channels during ischemia-reperfusion and hyperkalemic exposure show promising results on the preservation of NO and EDHF-mediated endothelial function, which suggests the potential of targeting endothelial K^+^ and TRP channels for myocardial protection during cardiac surgery.

## 1. Introduction

Coronary circulation is of vital importance to myocardial perfusion. The vascular endothelium of coronary arteries has been identified as the important organ that locally regulates coronary perfusion and cardiac function by producing vasoactive substances. The compromised function of coronary endothelium during cardiac surgery contributes to the no- or low-reflow phenomenon that ultimately leads to myocardial dysfunction and jeopardizes postoperative cardiac performance. Ischemia-reperfusion (I-R) and the direct contact of coronary endothelium with hyperkalemic solutions during cardioplegic intervention both pose detrimental effects on coronary endothelial function. Ion channels, in particular, potassium (K^+^) channels and calcium- (Ca^2+^-) permeable channels in endothelial cells, are essential to the production and/or function of endothelium-derived vasoactive factors. This review addresses the role of K^+^ and Ca^2+^-permeable channels in endothelial function by focusing on the regulation of vascular tone and summarizes the findings of alterations of these channels under conditions related to cardiac surgery. The potential of targeting these channels for myocardial protection during cardiac surgery is also discussed from the viewpoint of endothelial protection.

## 2. Endothelial Dysfunction during Cardiac Surgery: Effect of I-R and Cardioplegic Exposure

Endothelium functions to counteract leukocyte adhesion and platelet aggregation to prevent inflammation and thrombosis and actively regulate vascular tone by producing vasoactive substances [1, 2]. During cardiac surgery and cardioplegic intervention, coronary vasculature is inevitably subjected to I-R and hyperkalemic exposure. A considerable body of research shows the susceptibility of vascular endothelium to I-R or hypoxia-reoxygenation (H-R) injury. I-R/H-R activates endothelial cells resulting in neutrophil-endothelium adhesion and inflammation [3]. Activation of endothelial contractile machinery during I-R due to cell reenergization disturbs endothelial barrier function [4]. Moreover, I-R disrupts the balance between endothelium-derived constricting and relaxing factors and thus interrupts blood flow and organ perfusion [5]. The association of I-R and endothelial cell injury in cardiovascular surgery has been discussed in a previous review article by Boyle Jr and colleagues [6].

Cardioplegic and organ preservation solutions were initially designed to protect cardiac myocytes from I-R injury in heart surgery including heart transplantation. However, since endothelial cells differ with myocytes in structure, function, and electrophysiological properties (nonexcitable versus excitable), use of these solutions may not be able to provide protection to coronary endothelium. In fact, although there were studies showing the preservative effect of crystalloid cardioplegic or organ preservation solutions on endothelial function [7, 8], accumulating evidence suggests endothelial damage after exposure to these solutions. Histological examination and cell culture studies showed that crystalloid hyperkalemic cardioplegia impairs vascular endothelium and reduces the replicating ability of coronary endothelial cells [9, 10].

## 3. Impact of Cardioplegic Intervention on Endothelium-Derived Vasoactive Factors

Cardioplegic intervention interrupts the balance between endothelium-derived constricting and relaxing factors. I-R/H-R increased the production of vasoconstrictors such as endothelin-1 [5]. A large number of studies have revealed the significance of reduction of endothelium-derived relaxing factors (EDRFs), in particular, nitric oxide (NO) and endothelium-derived hyperpolarizing factor (EDHF), in the disturbance of blood flow in cardiac surgery-related conditions. Mechanisms underlying the impairment of endothelium-dependent vasorelaxation include I-R-induced availability and functional changes of NO and EDHF [11–16] as well as EDHF alterations caused by hyperkalemic exposure [17–20].

### 3.1. Impact of Cardioplegic Intervention on NO: Role of I-R and Hyperkalemic Exposure

Endothelial function mediated by NO, the major EDRF [21], is impaired during cardiopulmonary surgery. After 1-hour crystalloid cardioplegic arrest, NO release decreased significantly in human coronary vasculature and further decreased upon reperfusion, evidenced by the reduction of NO end-products nitrite and nitrate [22]. Inhibition of NO release after infusion of University of Wisconsin (UW) solution is associated with an attenuated endothelium-dependent vasodilatation [23]. Downregulation of eNOS protein was reported to underlie the loss of NO production caused by cardioplegia-reperfusion [24] and the NO loss after cold ischemic storage in crystalloid cardioplegia could be recovered by chronic oral administration of the NO substrate* L*-arginine [25]. All these studies demonstrated the unfavorable effect of cardioplegic intervention on endothelial eNOS-NO function. Consistently, we observed NO loss-related endothelial dysfunction and protection of endothelial function provided by eNOS enhancement in porcine coronary arteries which underwent 1 h of hypoxic exposure in St. Thomas cardioplegic (ST) solution and 30 min reoxygenation [26].

#### 3.1.1. I-R and NO

Endothelial I-R injury is closely associated with decrease of NO bioavailability. Myocardial I-R impairs endothelium-dependent NO-mediated relaxation in coronary arteries [27, 28]. Administration of* L*-arginine preserves postischemic endothelial function in both animals and humans [29, 30]. With measurement of NO using a NO microsensor, we provided direct evidence of NO reduction in coronary arteries in an* in vitro* I-R model [31].

In addition to the potent vasodilatory effect, NO inhibits platelet aggregation and leukocyte adhesion that is an important component of the endogenous defense mechanism against vascular injury, inflammation, and thrombosis. The loss of NO after myocardial I-R therefore poses profound deleterious effects on the coronary vasculature that further compromises myocardial function.

#### 3.1.2. Hyperkalemic Exposure and NO

Compared to numerous studies of I-R, investigations regarding the impact of hyperkalemic exposure* per se* on NO function remain limited. We reported that after exposure to crystalloid cardioplegia containing 16–50 mmol/L K^+^, endothelium-dependent vasorelaxations are well preserved in porcine epicardial coronary arteries [32] and neonatal rabbit aorta [33]. In these studies, the cardioplegic solutions were continuously oxygenated and the whole experiment was performed under well-oxygenated condition; therefore, the effect of I-R was completely excluded. It is worth mentioning that, in these studies, endothelium-dependent relaxation was studied in the presence of PGI_2_ inhibitor indomethacin; thus, the relaxation involves both NO and EDHF components. However, considering the susceptibility of EDHF to hyperkalemic exposure [17–19], which is addressed in details in the next section, the preserved endothelium-dependent dilator response to a certain extent suggests that hyperkalemic cardioplegic solutions may barely impair NO-related function. Direct measurements of NO demonstrated that NO production in porcine coronary arteries was unaffected by 1 h of exposure to hyperkalemic solution containing 20 mmol/L K^+^ [20]. Whether higher K^+^ concentration and longer exposure may affect endothelial NO production, however, remains unknown.

### 3.2. Impact of Cardioplegic Intervention on EDHF: Role of I-R and Hyperkalemic Exposure

The contribution of EDHF in vasodilatation increases as vessel size decreases [34, 35], which highlights the importance of this factor in blood flow regulation.

#### 3.2.1. I-R and EDHF

I-R alters EDHF-mediated endothelial function. In a rat I-R model of 2 h occlusion of the middle cerebral artery followed by 24 h reperfusion, the EDHF-mediated dilatation was potentiated [36, 37]. Potentiation of the EDHF-type response was also observed in dog coronary arteries subjected to 1 h of ischemia and 2 h of reperfusion [38]. These findings support the “compensatory or back-up” theory of EDHF mechanism in conditions involving NO loss. However, contradictory evidence is also available showing the deleterious effect of I-R on EDHF-mediated function. For example, in porcine coronary arteries exposed to H-R, the EDHF-mediated relaxation and hyperpolarization were significantly attenuated [15, 39–41]. H-R also blunted the EDHF response in coronary microveins [16]. The reasons of the controversy remain unclear that may include differences in species and vascular beds and differences in experimental settings. The residual NO may also have influence on the result interpretation in studies that only used eNOS inhibitor with no further use of NO scavenger to completely abolish the NO component.

#### 3.2.2. Hyperkalemic Exposure and EDHF

Our laboratory has contributed a great deal to the knowledge of the effect of hyperkalemia on EDHF pathway. With exclusion of the effect of I-R and elimination of the role of PGI_2_ and NO, we have demonstrated that hyperkalemic solutions [17–19] and clinically used crystalloid cardioplegia/organ preservation solutions such as ST [42] and UW solutions [43] impair EDHF-mediated function in porcine or human coronary arteries and veins. The depolarizing effect of hyperkalemia on the membrane of vascular smooth muscle cells counteracts and restricts the hyperpolarizing effect of EDHF, which was suggested to be responsible for the attenuated EDHF-mediated vasorelaxation under hyperkalemic cardioplegic exposure [17, 44]. This mechanism also explains the superiority of hyperpolarizing cardioplegia to depolarizing cardioplegia in preservation of EDHF-mediated endothelial function [45].

## 4. Role of Endothelial Ion Channels in NO and EDHF Pathways

Calcium- (Ca^2+^-) permeable and potassium (K^+^) channels are essential to vascular endothelial function. The importance of Ca^2+^-permeable channels is highlighted by the Ca^2+^-dependency of the synthesis or action of vasoactive agents including relaxing factors. Enzymes responsible for the production of NO and PGI_2_ requires an increase in endothelial [Ca^2+^]_i_ for activation [46, 47]. The classical EDHF response is initiated by opening of intermediate and small conductance Ca^2+^-activated K^+^ channels (IK_Ca_ and SK_Ca_) on the plasma membrane of endothelial cells [48]. In some vasculatures, nonclassical EDHF response mediated by epoxyeicosatrienoic acids (EETs) may also exist. EETs not only activate endothelial IK_Ca_ and SK_Ca_ but also open myocyte large-conductance K_Ca_ (BK_Ca_) to relax vessels [49]. The opening of IK_Ca_ and SK_Ca_ and the production of EETs also depend on [Ca^2+^]_i_ rise in endothelial cells [48, 50].

### 4.1. Ca^**2+**^-Permeable TRP Channels

Transient receptor potential (TRP) channels are the most important Ca^2+^-permeable channels in vascular endothelium [51–53]. TRP channels regulate [Ca^2+^]_i_ by directly acting as Ca^2+^ entry channels in the plasma membrane or by changing membrane potentials to modulate the driving force for Ca^2+^ entry [54]. Available evidence suggests that, among the six subfamilies of TRP (TRPA, TRPC, TRPV, TRPM, TRPP, and TRPML), canonical and vanilloid TRP (TRPC and TRPV) channels are more significantly involved in the vascular tone regulation. The role of TRPC1, C3, and C4 in Ca^2+^ influx in aortic endothelial cells has been implicated in the relaxant response of mouse aorta to agonists such as carbachol and acetylcholine [55, 56]. The partial involvement of TRPC6 in carbachol-induced relaxation was also observed in the mouse aorta [57]. TRPC3 forms channels endowed with significant constitutive activity [58] and native TRPC3 contributes to constitutive and ATP-dependent Ca^2+^ influx in human coronary artery endothelial cells [59]. Studies of various vascular beds including mesenteric [60], coronary [31, 59], and human internal mammary arteries, a widely used coronary arterial bypass graft [61], have revealed the significance of native TRPC3 in endothelial Ca^2+^ influx and vasorelaxation. Further mechanistic studies demonstrated that TRPC3-mediated Ca^2+^ influx in endothelial cells is necessary to the production of NO and the function of EDHF [31, 62, 63].

Participation of members of TRPV subfamily, in particular V4, was also reported in the control of vascular tone. In TRPV4-deficient mice, endothelium-dependent relaxations in response to acetylcholine and sheer stress were significantly attenuated in small resistance arteries, which was attributed to the inhibition of both NO and EDHF components [64, 65]. In fact, Ca^2+^ influx through TRPV4 has been proposed as a molecular mechanism sensing shear stress that significantly contributes to endothelial mechanotransduction [66]. Recently, in human coronary arterioles, Bubolz and colleagues demonstrated that TRPV4-mediated Ca^2+^ entry is involved in flow-induced vasodilatation. The TRPV4-dependent vasodilator response to arachidonic acid, a potentially important mediator of endothelium-derived hyperpolarizing-related vasodilation, was also reported [67, 68]. The findings that activation of merely a few TRPV4 channels enables local Ca^2+^ signals and causes maximal dilation through IK_Ca_ and SK_Ca_ activation further highlighted the functional significance of endothelial TRPV4 in vasorelaxation [69]. The physical interaction of TRPV4 with SK_Ca_ (K_Ca_ 2.3) in endothelial cells provided a structural basis for K_Ca_ activation by TRPV4-Ca^2+^ influx [70]. It is suggested that TRPV4-mediated [Ca^2+^]_i_ rise in endothelial cells may trigger both NO- and/or EDHF-dependent vasodilatation that seems to be vascular bed-dependent.

### 4.2. K^+^ Channels

Vascular endothelium expresses a variety of K^+^ channels including inwardly rectifying K^+^ (Kir), ATP-sensitive K^+^  (K_ATP_), voltage-gated K^+^  (K_V_), and Ca^2+^-activated K^+^  (K_Ca_) channels. The functional significance of Kv channels in endothelium physiology has not been established. Kir channels may serve as sensors for elevated extracellular K^+^ and contribute to K^+^-induced dilation [71] and act as amplifiers of hyperpolarization initiated by the opening of other K^+^ channels, in particular, IK_Ca_ and SK_Ca_, to conduct hyperpolarizing signals along endothelial cells [72]. In comparison with Kv and Kir, a considerable body of knowledge has been gained concerning the significance of K_ATP_ and K_Ca_ channels in endothelial function.

#### 4.2.1. K_ATP_ Channel

Patch-clamp studies in knockout mice suggested that endothelial K_ATP_ channels are an important component of shear-sensing mechanism in pulmonary microvasculature [73]. Shear stress increases the expression and activity of K_ATP_ channels in pulmonary vascular endothelial cells [74]. Previous studies also implicated the functional role of endothelial K_ATP_ channels in coronary vasodilatation, although evidence gained largely relied on the effect of pharmacological tools (channel antagonist or/and agonists) that therefore might be indirect. Endothelial K_ATP_ channels participate in flow and shear stress-mediated vasodilation [75] and dilations induced by isoflurane [76] and hyperosmolarity [77] in coronary microvessels. In the heart, K_ATP_ channels also expressed in endothelial cells of aorta and capillaries [78, 79]. It is believed that hyperpolarization mediated by K_ATP_ activation may facilitate Ca^2+^ influx in endothelial cells and thus modulate the production of EDRFs.

#### 4.2.2. K_Ca_ Channel

Endothelial IK_Ca_ and SK_Ca_ channels are of great importance in vascular tone regulation [80–83]. High or low SK_Ca_ expression in arteries of SK3 transgenic mouse, respectively, exaggerated or abolished the tonic endothelial dilating influence [84]. In IK_Ca_ knockout mice, disruption of the K_Ca_ 3.1 gene reduced acetylcholine-induced hyperpolarization in vascular cells which was associated with impairment of vasodilatation and elevation of blood pressure [85]. Intraluminal application of inhibitors of IK_Ca_ and SK_Ca_ channels blocked EDHF-mediated vasorelaxation [86]. Endothelial membrane hyperpolarization resulting from IK_Ca_ and SK_Ca_ opening can be conducted along the endothelium via homocellular endothelial gap junctions and transmitted to smooth muscle cells through myoendothelial gap junctions to cause vasodilatation. IK_Ca_ predominantly resides in myoendothelial projections whereas SK_Ca_ preferentially is located the sites of homocellular endothelial gap junctions and caveolin-rich domains [87–90]. The difference between the subcellular localization of these two subtypes is believed to be a mechanism facilitating efficient signaling transduction within endothelium and between endothelium and smooth muscle. Activation of IK_Ca_ and SK_Ca_ channels may also induce K^+^ efflux from endothelial cells that elicits hyperpolarization and relaxation of smooth muscle by activating Kir channels and Na^+^-K^+^-ATPase on the smooth muscle membrane [91]. In addition to the significant role in EDHF pathway [82, 83, 86–88, 92–94], activation of IK_Ca_ and SK_Ca_ channels may also amplify NO production in endothelial cells [95]. It was proposed that the channel opening-induced endothelial hyperpolarization enhances driving force for Ca^2+^ entry, resulting in elevation of [Ca^2+^]_i_ and amplification of NO production. The capacity of K_Ca_ channels in endothelial [Ca^2+^]_i_ modulation, therefore, further highlights the significance of these channels in the regulation of vascular tone.

## 5. Impact of Cardioplegic Intervention on K^+^ and TRP Channels in Endothelial Cells: Role of I-R and Hyperkalemic Exposure

### 5.1. Effect of I-R and Hyperkalemic Exposure on K_Ca_ Channels

Inactivation of IK_Ca_ and/or SK_Ca_ channels may contribute to endothelial dysfunction related to cardioplegic arrest/cardiopulmonary bypass in human. Following cardioplegic arrest/cardiopulmonary bypass, coronary arterioles and skeletal muscle arterioles showed significant decreases in vasorelaxant responses to IK_Ca_/SK_Ca_ activator and endothelium-dependent vasodilators [96, 97]. In these studies, the alteration of IK_Ca_ and SK_Ca_ occurred at channel activity level but not at expression level. Alteration of K_Ca_ channels under these circumstances may be attributable to both I-R injury and hyperkalemic cardioplegic exposure. Although there are controversial findings regarding the effect of I-R on K_Ca_ [98, 99], I-R-induced K_Ca_ inhibition was reported repeatedly. Vasorelaxant response to ADP significantly attenuated in goats underwent 1 h occlusion of the left middle cerebral artery followed by 1 h reperfusion and the loss of K_Ca_-EDHF mediation in postischemic vessels was evidenced by the loss of inhibitory effect of IK_Ca_ and SK_Ca_ channel blockers [100]. Consistently, we showed that EDHF-mediated hyperpolarization and relaxation were impaired in both coronary arteries and veins after 1 h hypoxia and 30 min reoxygenation [15, 16]. Further patch-clamp experiments demonstrated that inhibition of IK_Ca_ and SK_Ca_ channels is responsible for the compromised EDHF responses after H-R [41].

The effect of hyperkalemia on K_Ca_ channels remains poorly studied. We previously demonstrated that the IK_Ca_ and SK_Ca_-dependent EDHF responses are impaired by crystalloid hyperkalemic cardioplegia/organ preservation solutions in both coronary and pulmonary vasculatures [101–104]. Although these are not direct evidence of the inhibitory effect of hyperkalemia on endothelial K_Ca_ channel activity, given that K_Ca_ activation requires [Ca^2+^]_i_ rise and membrane depolarization in endothelial cells decreases the driving force for Ca^2+^ entry, suppression of K_Ca_ activity therefore may be expected when vascular endothelium is exposed to hyperkalemic cardioplegia/organ preservation solutions.

### 5.2. Effect of I-R and Hyperkalemic Exposure on K_ATP_ Channels

The findings of K_ATP_ mediation in the protection offered by preconditioning and postconditioning against endothelial I-R injury may suggest the association of K_ATP_ alterations and endothelial dysfunction [105–107]. Pharmacological inhibition of K_ATP_ channels reduced or completely blocked the protective effect of preconditioning and postconditioning on endothelium-dependent vasodilation. K_ATP_ channel blockade also abolished the protective effect of heat stress on endothelial function in ischemic heart [108]. K_ATP_ channel opener KRN_4884_ mimicked the protective effect of hypoxic preconditioning on the EDHF-mediated relaxation in coronary arteries [40], which may serve as an additional proof of I-R-induced functional disturbance of K_ATP_ channels. Further mechanistic investigations established the pivotal role of mitochondria K_ATP_ channels in cardioprotection. Opening of the mitochondrial K_ATP_ channel is thought to prevent the mitochondrial permeability transition pore from opening, leading to cellular protection from I-R injury in cardiac myocytes and vascular endothelial cells [109, 110].

Supplementation of K_ATP_ channel openers such as aprikalim and Nicorandil in hyperkalemic solutions protects EDHF-mediated function in coronary arteries [20, 111, 112]. Although the protection most likely results from the hyperpolarizing effect of K_ATP_ channel openers on smooth muscle membrane that counteracts hyperkalemia-induced depolarization thus facilitates EDHF responses, it cannot be ruled out that activation of K_ATP_ channels in endothelial cells might as well contribute to the protection of endothelial function. Endothelial K_ATP_ channel opening-induced membrane hyperpolarization may increase the driving force for Ca^2+^ influx thus promoting the production of EDRFs including EDHF.

## 6. Effect of I-R and Hyperkalemic Exposure on TRP Channels

I-R/H-R disturbs intracellular Ca^2+^ homeostasis and Ca^2+^ signaling in vascular endothelial cells. During ischemia, hypoxia/anoxia occurs in conjunction with acidosis and reperfusion is accompanied by increased formation of reactive oxygen species, which all have impact on [Ca^2+^]_i_. For example, [Ca^2+^]_i_ changes when extracellular acidosis occurs although the change may vary in different cells. Extracellular acidosis causes Ca^2+^ overload in anoxic endothelial cells from rat coronary artery [113], whereas in porcine aortic endothelial cells, extracellular acidification decreases [Ca^2+^]_i_ and inhibits agonist-stimulated production of EDRFs [114]. Suppression of store-operated Ca^2+^ entry via nonselective cation channels was found to be responsible for the decreased Ca^2+^ response in this study [114]. The modulation of store-operated Ca^2+^ entry was also reported in rat cardiac microvascular endothelial cells exposed to H-R [115]. Although these studies did not address by what molecular mechanisms store-operated Ca^2+^ entry is affected, they provided a logical basis for further investigation on the role of TRP channels in I-R-induced [Ca^2+^]_i_ disturbance given the significance of TRP channels in store-operated Ca^2+^ entry [52]. In fact, recent studies started to reveal the effects of I-R/H-R on TRP channels and associated vascular endothelial function. In patch-clamp studies of porcine coronary endothelial cells, we, for the first time, demonstrated that H-R suppresses TRPC3 channel activity through inhibiting the membrane translocation of this channel. The inhibition of TRPC3-mediated Ca^2+^ influx is an underlying mechanism of H-R-induced reduction of NO production and attenuation of endothelium-dependent relaxation in coronary arteries [31]. In a mice model of prolonged H-R, preconditioning amplifies EDHF-mediated relaxation with an association of an increase in TRPV4 expression in endothelial cells. Moreover, TRPV4 is involved in eNOS regulation in preconditioning, evidenced by an increase of phosphorylation on eNOS serine 1177 in wide type whereas an inhibition in TRPV4 knockout mice [116].

Changes of membrane potential affects [Ca^2+^]_i_. In endothelial cells depolarized by high K^+^ concentration, reduction of Ca^2+^ influx was observed [117, 118]. However, the mechanism by which endothelial Ca^2+^ channel is affected by hyperkalemia remains poorly studied. Our recent attempt in exploring the impact of hyperkalemia on endothelial Ca^2+^ channels provided the first evidence of the alteration of TRPC3 channels and the functional significance of TRPC3 alteration in hyperkalemia-induced EDHF dysfunction. We demonstrated that Ca^2+^ influx via TRPC3 channels in endothelial cells is reduced by hyperkalemia and activation of TRPC3 restores Ca^2+^ influx and prevents EDHF dysfunction caused by hyperkalemic solutions and clinically used crystalloid cardioplegia/organ preservation solutions such as ST and UW solutions [62].

## 7. Potential of Targeting K^+^ and TRP Channels for Endothelial Protection during Cardiac Surgery

Advances in understanding the mechanisms of endothelial dysfunction promote the development of strategies for endothelial cell protection. Better preservation of coronary endothelial and myocardial function can be achieved by supplementation of additives in cardioplegic or organ preservation solutions. Earlier studies have provided substantial evidence of the beneficial effect of supplementation of NO precursor* L*-arginine or NO donors, that is, nitroglycerin in cardioplegia on postischemic ventricular performance and endothelial function [119–121]. The EDHF component is well preserved in cardioplegic solutions containing EET_11,12_, a possible chemical candidate of EDHF [102, 122].

Earlier attempts including K_ATP_ channel openers in hyperkalemic cardioplegia have set a successful example of targeting K^+^ channels for endothelial protection. Hyperkalemia-induced impairment of EDHF function is ameliorated by addition of K_ATP_ channel openers such as nicorandil, aprikalim, and KRN_4884_ in cardioplegic solutions [15, 40, 112]. In addition, cardiac myocytes also benefit from the addition of K_ATP_ channel openers. Aprikalim inhibits Na^+^-Ca^2+^ exchange enhanced by hyperkalemia thus preventing [Ca^2+^]_i_ overload and improves the contractile function of ventricular myocytes [123]. Addition of the mitochondrial K_ATP_ opener diazoxide to heart preservation solution preserves diastolic function and reduces myocardial edema [124]. As to the benefit of K_ATP_ channel openers on endothelial function, although hyperpolarization of the smooth muscle cell membrane, the “effector” of EDHF responses is believed to underlie the preservation of EDHF pathway; the activation of K_ATP_ channels in endothelial cells might also be involved.

The unveiling of the key role of K_Ca_ and TRP channels in endothelial function together with the findings of the alterations of these channels during I-R and hyperkalemic exposure provided scientific basis for the potential of targeting these channels for endothelial protection during cardiac surgery. In an* in vitro* I-R model, the loss of endothelium-dependent relaxation of coronary arteries can be prevented by activation of IK_Ca_/SK_Ca_ and TRPC channels [31, 41]. Pharmacological activation of IK_Ca_ and SK_Ca_ channels during H-R improves EDHF responses including relaxation and hyperpolarization [41]. A recent review article provided an update on the effectiveness of IK_Ca_/SK_Ca_ activators in the treatment of cardiovascular disorders through the improvement of endothelium-derived hyperpolarizations [125]. Meanwhile, the therapeutic potential of activation of endothelial K_Ca_ channels to enhance NO availability in conditions associated with NO loss was reviewed by Kerr and colleagues [126].

Recent investigations started to show the potential of targeting endothelial TRP channels for cardiovascular protection, although so far the number of studies remains quite limited. In coronary arteries subjected to H-R, activation of TRPC3 channels in endothelial cells was observed to restore NO production [31]. Most recently, we demonstrated that supplementation of the TRPC3 activator in crystalloid cardioplegia such as ST and histidine-tryptophan-ketoglutarate (HTK) solution preserves TRPC3-mediated Ca^2+^ influx in endothelial cells and improves EDHF-mediated relaxation of coronary arteries [62]. The findings of protective effects of TRPC3 activation on coronary endothelium under H-R and hyperkalemic exposure may therefore shed light on future development of TRPC3-targeting strategies for myocardial protection during cardiac surgery.

Despite advances in the research of endothelial TRP channels, there are still many issues to be resolved, for example, the functional significance of interactions among different TRP isoforms. Whether a pathological condition that inhibits one isoform exerts the same effect on other isoforms or leads to “compensatory” enhancement of the function of others? Whether pharmacological modulation of one isoform has impact on others and changes their role in endothelial function? Answers to these questions are required to fully understand the potential of targeting TRP channels for endothelial cell protection.

In summary, Ca^2+^-permeable TRP and K^+^ channels in endothelial cells are essential to the regulation of vascular tone. Coronary endothelial dysfunction occurs in cardiac surgery that is attributable to I-R injury and hyperkalemic exposure. The functional alteration of endothelial TRP and K^+^ channels during I-R and hyperkalemic exposure renders these channels potential targets for endothelial protection during cardiac surgery.
